# Correction: Structural and Functional Similarity of Amphibian Constitutive Androstane Receptor with Mammalian Pregnane X Receptor

**DOI:** 10.1371/journal.pone.0104594

**Published:** 2014-07-30

**Authors:** The PLOS ONE Staff

## Notice of Republication

This article was republished on June 30, 2014, due to some characters in the Abstract and byline being erroneously changed during the typesetting process. The publisher apologizes for the errors. Please download this article again to view the correct version. The originally published, uncorrected article and the republished, corrected article are provided here for reference.

## Supporting Information

File S1 Originally published, uncorrected article.
(PDF)

File S2 Republished, corrected article.
(PDF)
