# Correction: Structural and Functional Similarity of Amphibian Constitutive Androstane Receptor with Mammalian Pregnane X Receptor

**DOI:** 10.1371/journal.pone.0148703

**Published:** 2016-02-01

**Authors:** Marianne Mathäs, Christian Nusshag, Oliver Burk, Ute Gödtel-Armbrust, Holger Herlyn, Leszek Wojnowski, Björn Windshügel

The second author’s name is spelled incorrectly. The correct name is: Christian Nusshag. The correct citation is: Mathäs M, Nusshag C, Burk O, Gödtel-Armbrust U, Herlyn H, Wojnowski L, et al. (2014) Structural and Functional Similarity of Amphibian Constitutive Androstane Receptor with Mammalian Pregnane X Receptor. PLoS ONE 9(5): e96263. doi:10.1371/journal.pone.0096263
