# Supplementary figures and images for: Structural and Functional Similarity of Amphibian Constitutive Androstane Receptor with Mammalian Pregnane X Receptor
Source: PLoS One. 2014 May 5;9(5):e96263. doi: 10.1371/journal.pone.0096263 (PMC4010427; doi:10.1371/journal.pone.0096263)

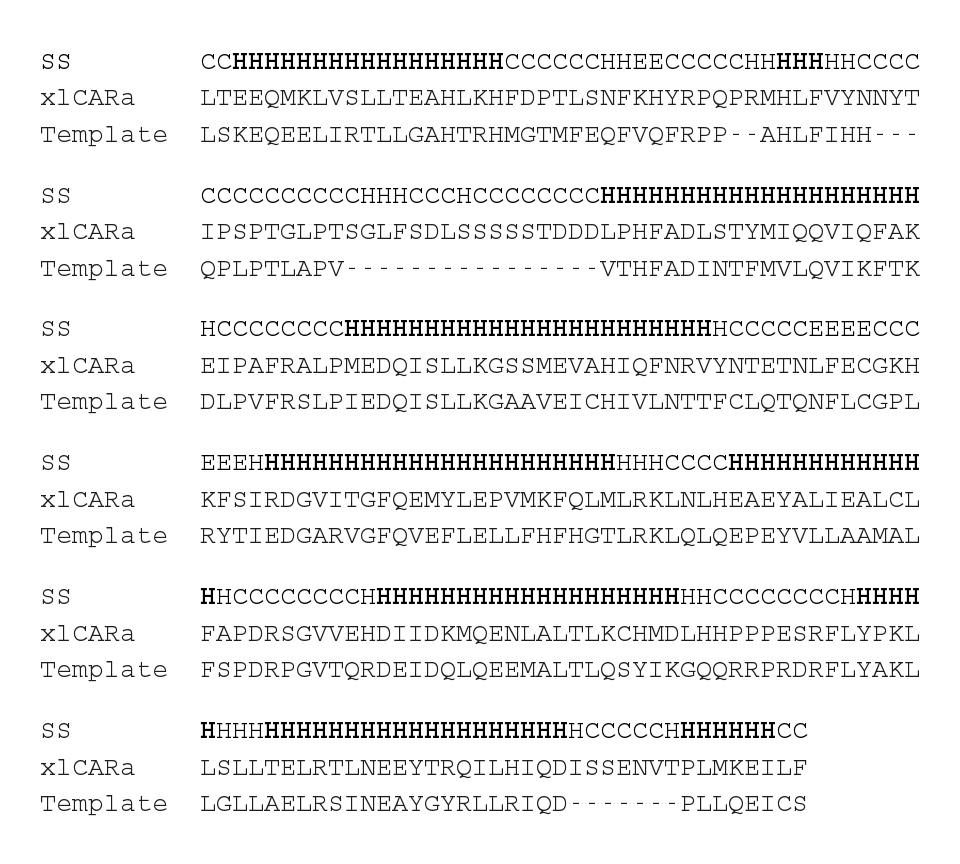

Supplement: Figure S1 — Sequence alignment of xlCARα template structure generated using CLUSTALW. Secondary structure prediction for xlCARα (H, helix; E, strand; C, coil) was performed using PSIPRED (http://bioinf.cs.ucl.ac.uk/psipred/). Predictions for single amino acids with a confidence level higher than 5 are highlighted in bold. (TIF) [file pone.0096263.s001.tif]

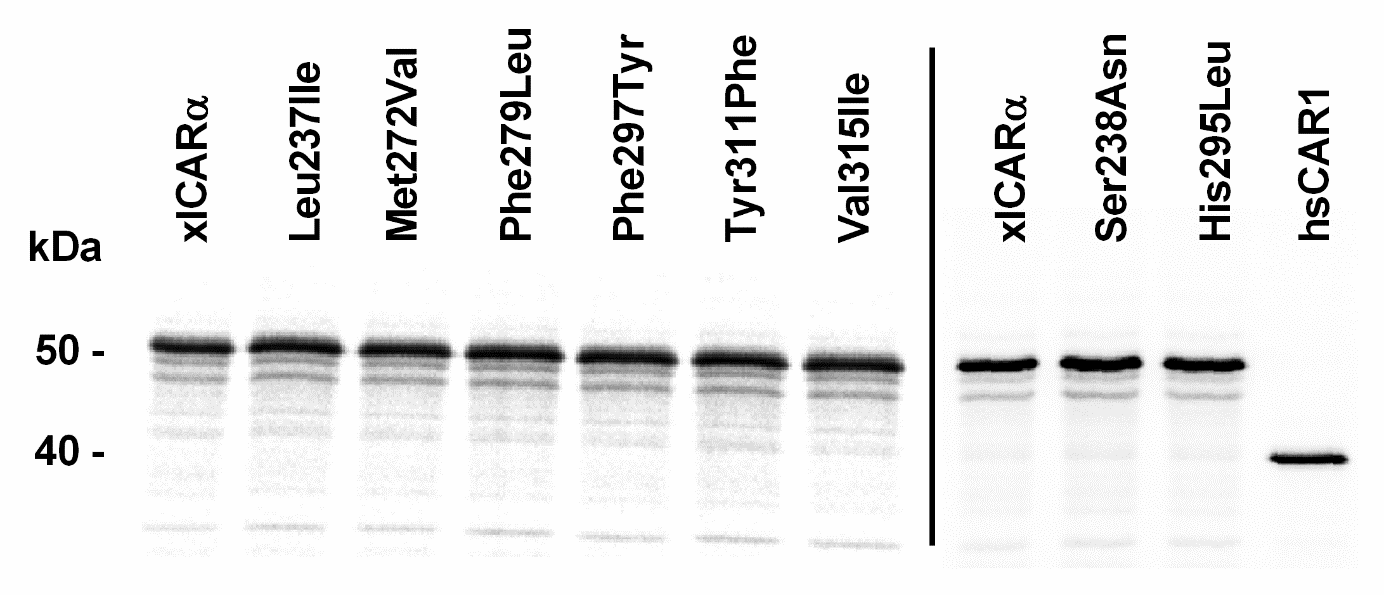

Supplement: Figure S2 — Equal expression of in vitro synthesized CAR proteins. Wild type and mutant xlCARα, and hsCAR1 proteins were labeled with 35S-methionine by in vitro transcription/translation using the respective expression plasmids. Aliquots of the reactions were analyzed by protein gel electrophoresis. (TIF) [file pone.0096263.s002.tif]

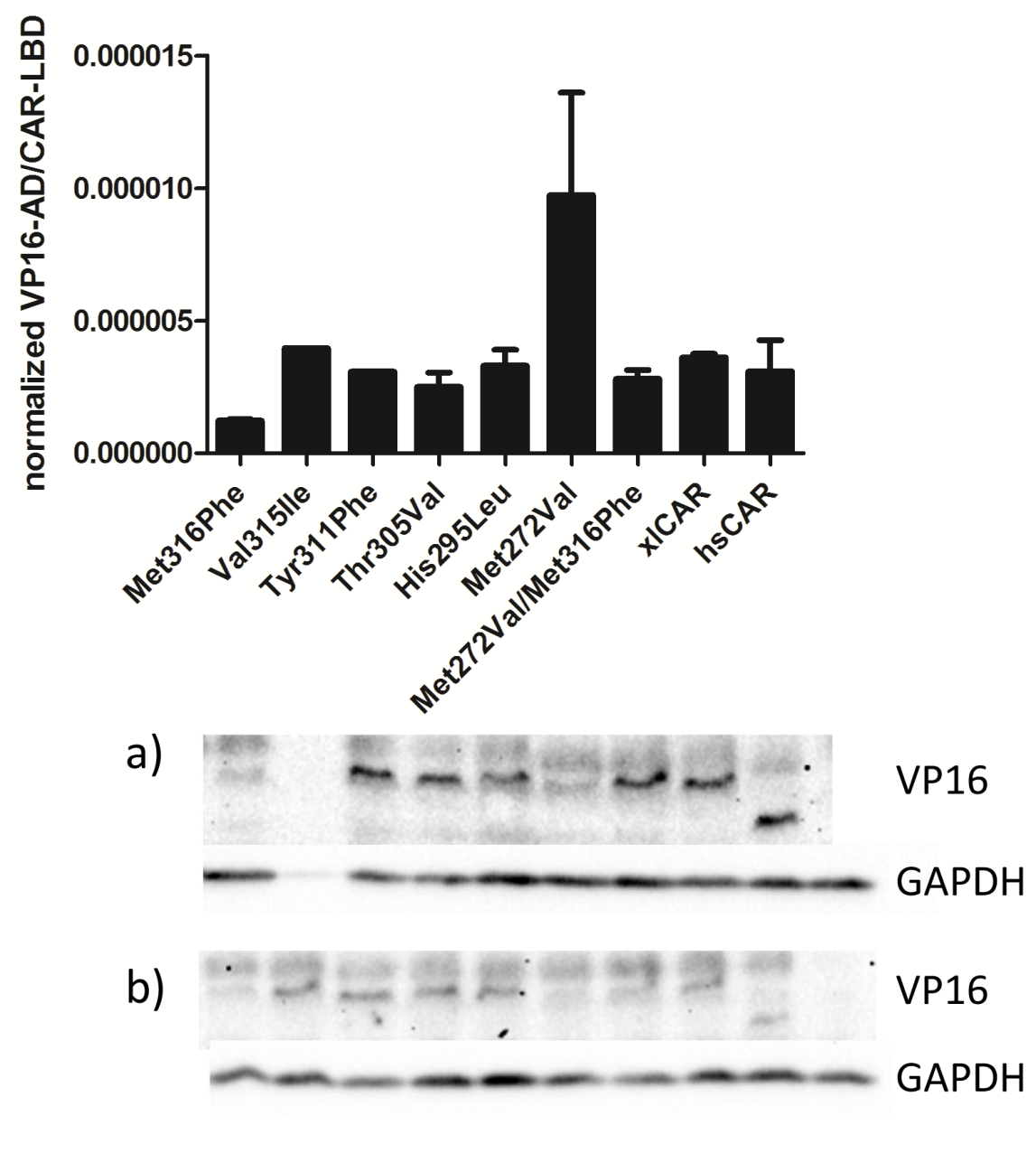

Supplement: Figure S3 — Expression levels of CAR-VP16 fusion proteins in COS 7 cells. Expression plasmids encoding fusion proteins of VP16-AD with wild type or mutant xlCARα or hsCAR LBD used in the mammalian two-hybrid assays were cotransfected with a Renilla luciferase reporter gene plasmid. Protein expression was analyzed in Western blots with anti-VP16 antibody and normalized for transfection efficiency via Renilla luciferase activity. The upper panel shows the respective quantification of VP16-AD/CAR-LBD fusion protein expression as mean ± SEM of 2 independent experiments. The two respective Western Blots are shown in a) and b). (TIF) [file pone.0096263.s003.tif]

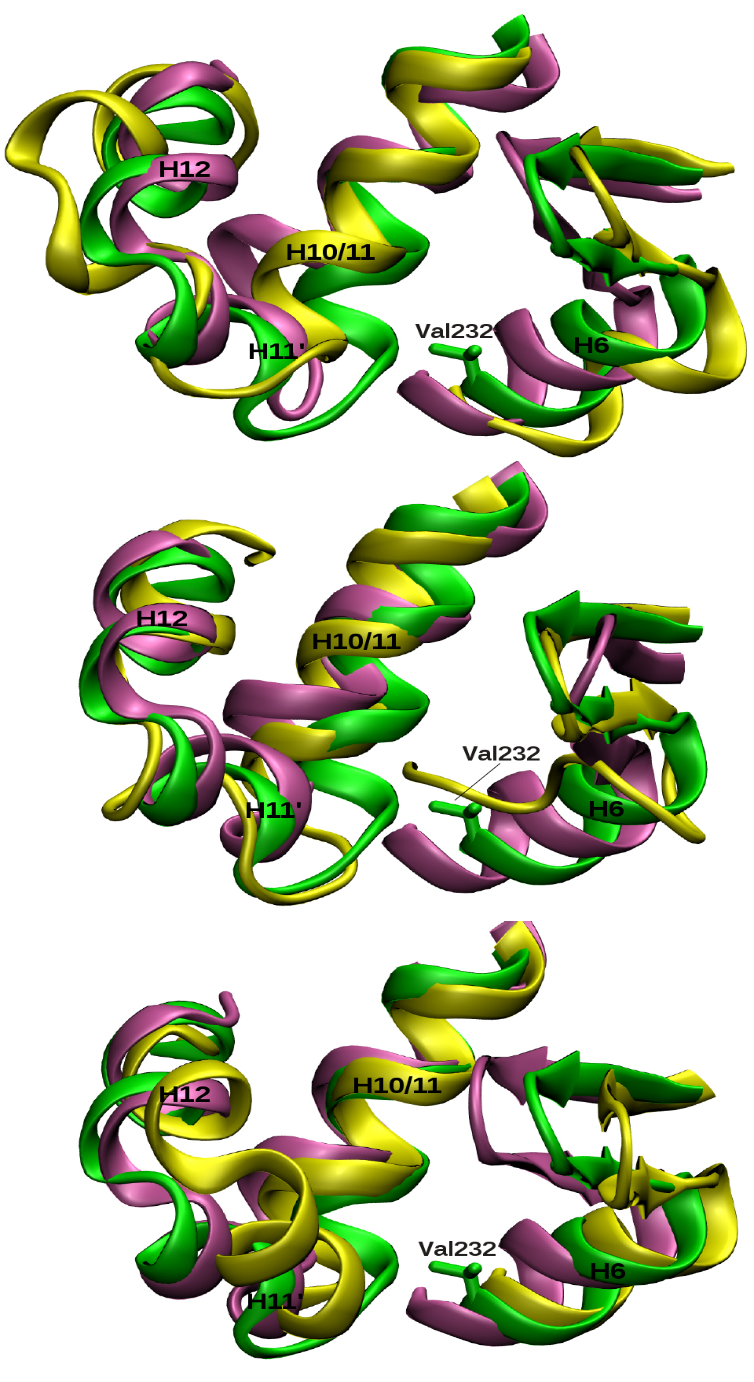

Supplement: Figure S4 — Conformational rearrangements within the ligand-binding domain of hsCAR upon the introduction of a Val232Thr mutation. Structures of wildtype (mauve) and mutant (yellow) receptor emerging after 50 ns of 3 different unconstrained molecular dynamics simulations are superimposed on the hsCAR x-ray crystal structure (PDB code 1xvp, colored in green). For the sake of clarity the structures are shown in ribbon representation and large parts of the LBD are excluded. The side chain of Val232 of the x-ray crystal structure is shown explicitly (green). (TIF) [file pone.0096263.s004.tif]
