# Supplementary material for: Structural and Functional Similarity of Amphibian Constitutive Androstane Receptor with Mammalian Pregnane X Receptor
Source: PLoS One. 2014 May 5;9(5):e96263. doi: 10.1371/journal.pone.0096263 (PMC4010427; doi:10.1371/journal.pone.0096263)
Supplement: Table S1 — Primers used for generation of point mutations and deletions within the hsCAR, xlCARα and hsPXR ligand-binding domains. (DOCX) [file pone.0096263.s005.docx]

**Table S1. Primers used for generation of point mutations and deletions within the hsCAR, xlCARα and hsPXR ligand-binding domains.**

| **Name** | **Sequence** |
| --- | --- |
| del xlCARα (556-687) fw | 5'-gatgcacctgttcgtatacgatttgccgcattttgctg-3' |
| del xlCARα (556-687) rv | 5'-cagcaaaatgcggcaaatcgtatacgaacaggtgcatc-3' |
| del hsPXR (544-699) fw | 5'-ggtgcttagcagtggcaaagagatcttctccc-3' |
| del hsPXR (544-699) rv | 5'-gggagaagatctctttgccactgctaagcacc-3' |
| xlCARα His295Leu fw | 5'-caatctctttgagtgtggaaaacttaaattcagcataagagatggtg-3' |
| xlCARαHis295Leu rv | 5'-caccatctcttatgctgaatttaagttttccacactcaaagagattg-3' |
| xlCARα Thr305Val fw | 5'-gaaaacataaattcagcataagagatggtgttatagttggattccaagagatgta-3' |
| xlCARα Thr305Val rv | 5'-tacatctcttggaatccaactataacaccatctcttatgctgaatttatgttttc-3' |
| xlCARα Met418Leu fw | 5' CTGAAAACGTCACCCCTCTGCTGAAAGAGATTCTATTC 3' |
| xlCARα Met418Leu rv | 5' GAATAGAATCTCTTTCAGCAGAGGGGTGACGTTTTCAG 3' |
| xlCARα Val315Ile fw | 5' GGATTCCAAGAGATGTTCCTGGAACCGGTGATG 3' |
| xlCARα Val315Ile rv | 5' CATCACCGGTTCCAGGAACATCTCTTGGAATCC 3' |
| xlCARα Leu422Cys fw | 5' CCTCTGATGAAAGAGATTTGCTTCTAATCTAGAGGGCCC 3' |
| xlCARα.Leu422Cys.rv | 5' GGGCCCTCTAGATTAGAAGCAAATCTCTTTCATCAGAGG 3' |
| xlCARα del Asp408+Ile409 fw | 5'-cagatccttcacattcagtcttctgaaaacgtcacc-3' |
| xlCARα del Asp408+Ile409 rv | 5'-ggtgacgttttcagaagactgaatgtgaaggatctg-3' |
| hsCAR insAsp+Ile after Gln334 fw | 5'-ccagcacatccaggatataggcctgtctgcca-3' |
| hsCAR insAsp+Ile after Gln334 rv | 5'-tggcagacaggcctatatcctggatgtgctgg-3' |
| xlCARα Asp413Pro fw | 5'-tcacattcaggatatatcttctgaacccgtcacccctctgatg-3' |
| xlCARα Asp413Pro rv | 5'-catcagaggggtgacgggttcagaagatatatcctgaatgtga-3' |
| hsCAR Ala338Pro fw | 5'-cacatccagggcctgtctcccatgatgcc-3' |
| hsCAR Ala338Pro rv | 5'-ggcatcatgggagacaggccctggatgtg-3' |
