# Supplementary material for: Correction: Structural and Functional Similarity of Amphibian Constitutive Androstane Receptor with Mammalian Pregnane X Receptor
Source: PLoS One. 2014 Jul 30;9(7):e104594. doi: 10.1371/journal.pone.0104594 (PMC4116230; doi:10.1371/journal.pone.0104594)
Supplement: File S1 — Originally published, uncorrected article. (PDF) [file pone.0104594.s001.pdf]

OPEN ACCESS Freely available online

PLOS ONE

Structural and Functional Similarity of Amphibian Constitutive Androstane Receptor with Mammalian Pregnane X Receptor

Marianne Mathäs[1], Christian Nuβhag[1], Oliver Burk[2], Ute Gödtel-Armbrust[1], Holger Herlyn[3], Leszek Wojnowski[1], Björn Windshügel[4,5]*

1 Department of Pharmacology, University Medical Center, Mainz, Germany, **2** Dr. Margarete Fischer-Bosch-Institute of Clinical Pharmacology, Stuttgart, Germany, **3** Institute of Anthropology, Johannes Gutenberg-University, Mainz, Germany, **4** Centre for Bioinformatics, University of Hamburg, Hamburg, Germany, **5** European ScreeningPort GmbH, Hamburg, Germany

Abstract

The nuclear receptors and xenosensors constitutive androstane receptor (CAR, NR1I3) and pregnane X receptor (PXR, NR1I2) induce the expression of xenobiotic metabolizing enzymes and transporters, which also affects various endobiotics. While human and mouse CAR feature a high basal activity and low induction upon ligand exposure, we recently identified two constitutive androstane receptors in Xenopus laevis (xlCARá and â) that possess PXR-like characteristics such as low basal activity and activation in response to structurally diverse compounds. Using a set of complementary computational and biochemical approaches we provide evidence for xlCARá being the structural and functional counterpart of mammalian PXR. A three-dimensional model of the xlCARá ligand-binding domain (LBD) reveals a human PXR-like L-shaped ligand binding pocket with a larger volume than the binding pockets in human and murine CAR. The shape and amino acid composition of the ligand-binding pocket of xlCAR suggests PXR-like binding of chemically diverse ligands which was confirmed by biochemical methods. Similarly to PXR, xlCARá possesses a flexible helix 11′. Modest increase in the recruitment of coactivator PGC-1á may contribute to the enhanced basal activity of three gain-of-function xlCARá mutants humanizing key LBD amino acid residues. xlCARá and PXR appear to constitute an example of convergent evolution.

Citation: Mathäs M, Nuβhag C, Burk O, Gödtel-Armbrust U, Herlyn H, et al. (2014) Structural and Functional Similarity of Amphibian Constitutive Androstane Receptor with Mammalian Pregnane X Receptor. PLoS ONE 9(5): e96263. doi:10.1371/journal.pone.0096263

Editor: Rolf Müller, Philipps University, Germany

Received December 6, 2013; **Accepted** April 4, 2014; **Published** May 5, 2014

Copyright: © 2014 Mathäs et al. This is an open-access article distributed under the terms of the Creative Commons Attribution License, which permits unrestricted use, distribution, and reproduction in any medium, provided the original author and source are credited.

Funding: The work was supported by the institutions employing the authors: University Medical Center, Mainz; Institute of Clinical Pharmacology, Stuttgart; Institute of Anthropology, Johannes Gutenberg—University Mainz; European ScreeningPort GmbH, Hamburg. The funders had no role in study design, data collection and analysis, decision to publish, or preparation of the manuscript.

Competing Interests: Currently, co-author Björn Windshügel is employed by the European ScreeningPort GmbH. This does not alter the authors' adherence to PLOS ONE policies on sharing data and materials.

* E-mail: bjoern.windshuegel@screeningport.com

Introduction

Ligand-modulated transcription factors of the nuclear receptor (NR) superfamily are involved in regulation of various physiological processes such as development and homeostasis and also play a prominent role in metabolism and disposition of exogenous compounds from the body [1], [2]. In mammals the latter process is mainly governed by pregnane X receptor (PXR, NR1I2) and constitutive androstane receptor (CAR, NR1I3). CAR and PXR arose in early vertebrates from a common ancestral gene (Figs. 2 and 4 in [3]) and they both recognize chemically diverse substances including pesticides, plasticizers as well as prescription drugs [4]. Target genes of CAR and PXR comprise two overlapping panels of phase I-III metabolizing enzymes and transporters. Represented by cytochrome P450 (CYP) enzymes CYP2B6 and CYP3A4, the most relevant human drug metabolizing CYPs are under transcriptional control of CAR and PXR [5].

In contrast to most other NRs, including PXR, the major splice variant of human CAR (CAR SV1) reveals a pronounced ligand-independent activity *in vitro* accompanied by a limited activation potential upon agonist binding [6], [7]. In contrast, the recently identified *Xenopus laevis* CARα (xlCARα) possesses a low basal activity *in vitro* and a large induction potential by agonists [3]. The ligand spectrum (including natural products, prescription drugs and endogenous substances) was found to be significantly broader compared to the human ortholog. Further investigations revealed that the constitutive CAR activity typical for mammals emerged first with fully terrestrial land vertebrates [3].

By means of experimental and theoretical studies, the structural basis for the constitutive activity of human and mouse CAR has been addressed in several previous studies. Using homology modeling approaches and X-ray crystallography studies, 3-dimensional structures of the CAR ligand-binding domain (LBD) have been generated and used for identification of single amino acids relevant for constitutive activity with subsequent experimental validation by means of site-directed mutagenesis [8–12]. In many cases the standard procedure – replacing the amino acid of interest by alanine – resulted in complete loss of basal activity and ligand responsiveness, thus demonstrating the limiting informative value of this approach. Recently, we identified two amino acids within the xlCARα ligand-binding pocket (LBP) whose mutation towards the human receptor resulted in a substantially increased

basal activity [3]. The gain in receptor activity was accompanied by a reduced ligand induction potential. The mutated receptor phenotypically resembled human CAR, providing a model system for further investigating the structural basis of its constitutive activity.

In addition, our recent study provided first evidence for xlCARα involvement in physiological processes different from those described previously for mammalian CAR as suggested by a much wider xlCARα organ expression. Besides liver, the receptor is strongly expressed in lung, skin, stomach, kidney and ovary, with lower expression levels in brain and heart [3]. This is in clear contrast to human CAR expression that – besides liver and intestine – is only expressed at low levels in other tissues [4]. Therefore, xlCARα inducers may be capable to affect more organs in amphibians, which may contribute to the extraordinary sensitivity of amphibians to environmental pollutants compared to mammalians [3]. A more detailed investigation of the structural and functional properties of amphibian constitutive androstane receptor may support future toxicological and ecological risk assessments. Moreover, as shown by the previously identified gain-of-function mutations, the receptor may represent a model system for further investigating the structural determinants of mammalian CAR basal activity.

In this study, we intended to characterize the xlCARα ligand-binding domain from a structural and functional point of view by investigating the overall LBD structure, its interaction possibilities with ligands and the heterodimerization partner RXRα (retinoid X receptor α) as well as two coactivators, using a combination of *in silico* and *in vitro* approaches. As no x-ray crystal structure is available for the xlCARα LBD, we utilized a homology modeling approach, similar to previous studies on human CAR [11–13], by which the three-dimensional structure of the xlCARα ligand-binding domain was constructed based on a sequentially related x-ray crystal structure. The model guided the selection of amino acids to be mutated in *in vitro* assays, validating the outcome of the modeling process. Putative binding modes of previously identified agonists were determined using a molecular docking approach. This procedure was validated and complemented by various *in vitro* assays using receptor mutants by which the effects of ligand binding on receptor activation and heterodimerization with RXRα as well as on recruitment of coactivators SRC-1 and PGC-1α were investigated.

Materials and Methods

Homology model generation

In order to generate a homology model of the xlCARα ligand-binding domain, a BLAST search querying the Protein Data Bank (PDB) was performed for identification of sequentially related protein structures. Subsequently, x-ray crystal structures of proteins with highest sequence similarity (human CAR and PXR) were downloaded from the PDB [14]. Similar to human VDR and PXR, xlCARα contains a H1–H3 insert [3]. According to secondary structure predictions using PSIPRED (http://bioinf.cs.ucl.ac.uk/psipred/, see also Figure S1), the xlCARα H1–H3 insert lacks any secondary structural elements. Thus, PXR x-ray crystal structures are not suited as template structures as these contain a helix and two β-strands within the H1–H3 which are associated with further structural changes in helices 6 and 7 [15].

The identification of a suitable template structure from the set of available CAR x-ray crystal structures was guided by stereochemical parameters as determined with PROCHECK and the folding reliability as assessed with ProSa 2003 [16], [17]. Finally, PDB code 1xv9 was chosen as main modeling template [9]. Including results from secondary structure prediction for xlCARα using PSIPRED, two modifications were introduced using MOE (Chemical Computing Group, Montreal, Canada): as helix 3 was predicted to be shorter in xlCARα than in human CAR, amino acids 155–157 were deleted from the original template structure. In return, PSIPRED predicted helix 10/11 to possess a C-terminal extension. Therefore, coordinates for residues 410–416 of human PXR (PDB code 1nrl), that contains an extended helix 10/11, were transferred into the template structure upon superpositioning of PXR with CAR. As protein modeling tools generally fail to reliably predict structures of loops longer than 10–12 residues, amino acids 199–230 of the insert were not considered for the homology modeling procedure. The homology model of xlCARα was constructed employing MOE thereby using the sequence alignment shown in Figure S1. Initially, ten structures were generated which were energy minimized using the Amber force field with Born solvation as implemented in MOE. Coordinates of the final protein model are available on request from the authors. Shapes of the ligand-binding pockets of xlCARα, hsCAR and hsPXR and determination of their volumes were calculated using MOLCAD within SYBYl-X1.2 (Tripos Int., St Louis, MO, USA) after addition of all hydrogen atoms.

Molecular Dynamics Simulations

Human CAR x-ray crystal structure (PDB code 1xvp, chain D) was used for the simulations. The Thr232 mutation was generated using SCWRL3 [18]. All simulations were performed using GROMACS version 3.3.3 [19]. The setup of the simulation system as well as the equilibration and production procedures were performed as described previously [20].

Molecular docking

Three-dimensional structures of known xlCARα ligands artemisinin, fenofibrate and pregnanedione were generated within SYBYL-X1.2 and subsequently energetically minimized. Molecular docking was performed using GOLD version 5.1 (Cambridge Crystallographic Data Centre). For each ligand, 10 independent docking runs were performed. The early termination option was switched off. Of the scoring options provided with GOLD, ChemScore was selected.

Plasmids and mutagenesis

The following firefly luciferase reporter gene plasmids have been described previously: human CYP3A4 enhancer/promoter plasmid pGL3-CYP3A4(7830/Δ7208–364) [21], hereinafter called CYP3A4 enhancer/promoter; human MDR1 enhancer DR4 (I) element-dimer/Tk promoter plasmid [22], hereinafter called DR4-Tk promoter; Gal4-dependent reporter gene construct pGL3-G5 [23]. The *Renilla* luciferase reporter gene plasmid pRL-EF1α1 was described previously [24]. The following eukaryotic expression plasmids have been published elsewhere: encoding full-length human CAR1 [25], xlCARα [3] and human PXR [22]; encoding fusion proteins of the GAL4 DNA-binding domain (DBD) and receptor interaction domain (RID) of human coactivator SRC-1 (NCOA1, amino acids 583–783) [23], or RID of human PGC-1α (amino acids 88-213) [3], or ligand binding domain (LBD) of human RXRα (amino acids 226–462) [23]; encoding fusion proteins of the VP16 activation domain (AD) and LBD of xlCARα (amino acids 145–423) [3], or LBD of human CAR1 (amino acids 105–348) [23].

Base pair mutations and deletions were introduced into the respective expression plasmids using appropriate oligonucleotide primers (Table S1) and the QuikChange Site-Directed Mutagen-

esis Kit (Agilent, Santa Clara, CA), according to manufacturer's instructions. All mutated constructs were verified by sequencing.

Cell culture, transient transfections and reporter gene assays

Human colon adenocarcinoma LS174T cells (ATCC, Manassas, VA) and human hepatoma HepG2 cells (ATCC) were maintained as previously described [23]. Cells were plated into 96-well plates at a density of 2.5×10^4 cells/well (LS174T) or 24-well plates at 1.5×10^5 cells/well (HepG2) 24 h before transfection, which was performed using GeneJuice (Merck Millipore, Darmstadt, Germany) or Effectene (Qiagen, Hilden, Germany) reagents for LS174T or HepG2 cells, respectively, according to manufacturer's instructions. The combinations of co-transfected firefly luciferase reporter gene and expression plasmids are indicated in the respective Figure legends. Firefly luciferase activities, determined in cell lysates as described [26], were normalized with respect to *Renilla* luciferase activities of co-transfected pRL-EF1α1. At least three independent experiments, each done in triplicates, were performed using a minimum of two different plasmid DNA preparations of the respective nuclear receptor expression plasmids.

For mammalian two-hybrid assays, COS7 cells were plated into 96-well plates at a density of 1,500 cells per well. Cells were transfected using Gene Juice and 36.7 ng of pGL3-G5, 3.3 ng of the respective GAL4-DBD-fusion protein expression plasmid, together with 26.7 ng of respective expression plasmids encoding fusion proteins of VP16-AD and the wild type or mutant xlCARα LBDs and 7.7 ng of pRL-EF1α1. Protein expression of the VP16-AD/CAR-LBD fusion proteins were determined by Western blot using anti-VP16 (14–5) antibody (Santa Cruz).

Recombinant protein expression

Recombinant human SRC-1 protein (amino acids 583–783, comprising the receptor interaction domain), fused at the amino-terminus to GST, was expressed in bacteria as described previously [27]. *In vitro* transcription/translation (TNT T7 quick coupled transcription/translation system, Promega, Madison, WI, USA) was used to synthesize ^{35}S-methionine labelled full-length X. laevis and human CAR proteins in 25 µl reactions, containing 0.5 µg of the respective expression plasmids and 10 µCi ^{35}S-methionine (specific activity 1175 Ci/mmol, radioactive concentration 10 µCi/µl, MP Biomedicals, Santa Ana, CA, USA). Equal protein expression of mutant receptors (Fig. S2) was confirmed by protein gel electrophoresis of an aliquot. Gels were stained with Coomassie, dried and exposed to BAS-IP MS2325 imaging plates (Fuji, Kanagawa, Japan), which were quantified using the CR35 Bio radioluminography laser scanner and AIDA software (Raytest, Straubenhardt, Germany).

Coactivator-dependent receptor ligand assay (CARLA)

CARLA was performed essentially as described before [27]. Briefly, 1 ml reactions were set up in NETN (100 mM NaCl, 1 mM EDTA, 1 mM DTT, 20 mM Tris-Cl pH 8.0, 0.5% (v/v) Nonidet P40), with 0.5% (w/v) skimmed milk powder, using 3–5 µg of GST-tagged SRC-1 RID protein, bound to 25 µl bead volume of glutathione-sepharose 4B beads (GE Healthcare, Freiburg, Germany), 2 µl of respective ^{35}S-labelled CAR protein, and the respective chemicals or 1% solvent DMSO only. After incubation over night at 4°C with constant rotation, beads were washed three times in 1 ml NETN buffer, supplemented with the respective chemicals. Bound GST/SRC-1 fusion protein/CAR complexes were extracted from the beads by boiling in SDS-protein sample buffer and separated on 10% SDS-polyacrylamide gels, which were subsequently stained with Coomassie, dried and exposed to BAS-IP MS 2325 imaging plates. CAR protein bound to SRC-1 was detected by reading the image plates with CR35 Bio radioluminography laser scanner and quantified by densitometric scanning of the image, using AIDA software. Coomassie staining of the protein gels demonstrated the use of equal amounts of GST/SRC-1 fusion protein in each reaction. Respective control experiments, which had been set up with GST protein only, demonstrated negligible binding of CAR proteins to the GST moiety of the GST/SRC-1 fusion protein.

Statistical analysis

Multiple comparisons were performed by one-way or two-way analysis of variance (ANOVA) with Dunnett's multiple comparisons test, as indicated. Pairwise comparisons were done by paired *t*-test and comparisons to a hypothetical mean by one sample *t*-test. In the latter case, the resulting *P*-values were corrected for multiple testing using the method of Bonferroni. All calculations were performed with GraphPad Prism version 6.03 (GraphPad Software, La Jolla, CA). Statistical significance was defined as $P<0.05$.

Results

Homology model of xlCARα

In order to investigate the 3-dimensional structure of *X. laevis* constitutive androstane receptor xlCARα, we constructed a homology model of its ligand-binding domain (residues 145–423). Utilizing sequence comparisons with related nuclear receptors and secondary structure predictions, a chimeric template structure for the homology modeling process was constructed from coordinates of x-ray crystal structures solved for human CAR and PXR.

As no suitable template structure was available for modeling the 62 residues long and, according to results from secondary structure prediction tools, unstructured H1–H3 insert, this part of the LBD was largely excluded (amino acids 199–230) from the modeling process. In order to justify this approach we experimentally assessed this region for any involvement in receptor activity or ligand-binding as seen in x-ray crystal structures of human PXR [15]. At first, a deletion mutant excluding amino acids 186–229 of xlCARα was generated. For further comparison with PXR, which possesses a similar H1–3 insert contributing to LBP-formation, the corresponding region (residues 182–233) was removed. While the PXR deletion mutant resulted in a loss-of-function, the truncated xlCARα revealed no change in basal activity and responsiveness to ligands (Fig. 1), suggesting that the H1–H3 insert is not involved in LBP formation.

The stereochemical quality of the resulting protein model was evaluated using PROCHECK and ProSa. The Ramachandran plot revealed 97.8% of all amino acids adopting most favorable or additionally allowed φ/ψ backbone torsion angles. Residues with less favorable geometries were found to be largely located in the remaining parts of the H1–H3 insert. The ProSa Z-score of -10.28 for the 247 amino acids comprising xlCARα LBD indicated a reliable overall fold and was better than all scores determined for human CAR x-ray crystal structures (varying from −9.70 to −10.05) which possess an almost identical number of residues. Protein regions with unfavorable energies were mainly restricted to the truncated H1–H3 insert (data not shown).

Figure 2A shows the three-dimensional structure of the resulting LBD homology model. Due to the small extension of helix 10/11 at its C terminus, the xlCARα model suggests the absence of a

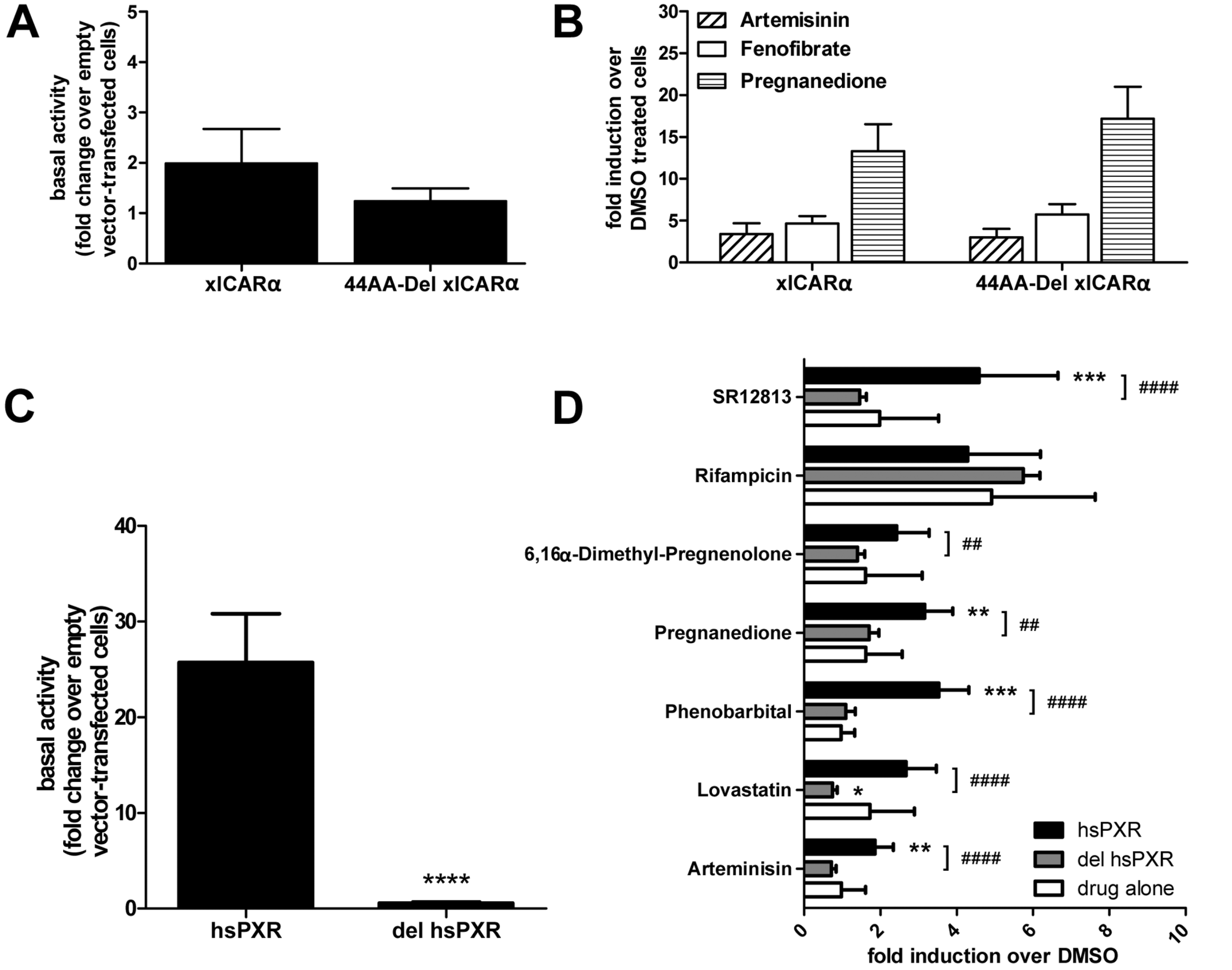

Figure 1. Role of the H1-H3 interhelical domain for X. laevis CARα and human PXR activity. Comparison of basal (A) and ligand-dependent activities (B) of wildtype xlCARα (xlCARα) and xlCARα with deletion of amino acids 186–229 (44AA-Del xlCARα). LS174T cells were co-transfected with expression plasmids encoding the indicated proteins and the CYP3A4 enhancer/promoter reporter gene plasmid. Comparison of constitutive (C) and ligand-dependent (D) activities of wildtype hsPXR (hsPXR) and its mutant missing AS 182 to 233 (del hsPXR). LS174T cells were co-transfected with respective expression plasmids and a Cyp2B10 promoter element driving luciferase expression. Ligands were used at 10 µM with the exception of artemisinin (100 µM), lovastatin (30 µM) and phenobarbital (1 mM). Data are presented as means ± SEM (n = 6 independent experiments), with activity in the presence of empty expression vector pcDNA3 only (A,C) or of treatment with vehicle DMSO only (B,D) set as 1. Statistically significant differences between wild type and mutant proteins, as determined by t-test (A,C) or one-way ANOVA with Dunnett's multiple comparisons test (B,D), are indicated by asterisks. *, $P<0.05$; **, $P<0.01$; ***, $P<0.001$, no asterisks indicate no differences.
doi:10.1371/journal.pone.0096263.g001

helical segment in between helix 10/11 and the activation helix (H12) found in x-ray crystal structures of murine and human CAR. Instead, the loop shows an extended conformation.

The L-shaped ligand-binding pocket is framed by 33 amino acids located on helices 3, 5–7, 10 and 12 as well as β-strands 1–3 (Table 1). Additionally, few amino acids of the undeleted parts of the H1–H3 insert were found to line the LBP. However, as a major part of the insert was excluded from the modeling procedure, any contribution of those residues to LBP formation remains speculative and thus they are not listed in Table 1. The ligand-binding pocket captures a volume of 915 $Å^3$ which is larger compared to human and murine CAR (750–810 $Å^3$), but smaller than in human PXR (1320–1420 $Å^3$). The shape of the pocket less resembles the ellipsoid shape of human or mouse CAR and is more alike the T-shaped form observed in human PXR x-ray crystal structures (Fig. 2B&C).

Similar to human CAR, the LBP is almost completely separated from the activation helix by a barrier formed by amino acids Phe234, Ser238 and Ile242 (all on H3), Met272 (on H5) as well as by Tyr399 and Ile403 (both on H10/11). A hydrogen bond is shared between Ser238 and Tyr399 side chains, connecting helices 3 and 10/11. A similar interaction is seen in mammalian CAR, involving Asn165 (H3) and Tyr326 (H10/11) of the human receptor.

In hsCAR, a hydrogen bond connects Tyr224 on the $β_3$ strand and His160 (H3). This interaction cannot be established in xlCARα as the corresponding amino acids (Phe297 and His233) do not allow hydrogen bond formation between their side chains. Instead, the model suggests a hydrogen bond between His295 and His233.

As indicated by the lipophilic potential mapped on the surfaces of the ligand-binding pockets in Figure 2, the xlCARα LBP amino

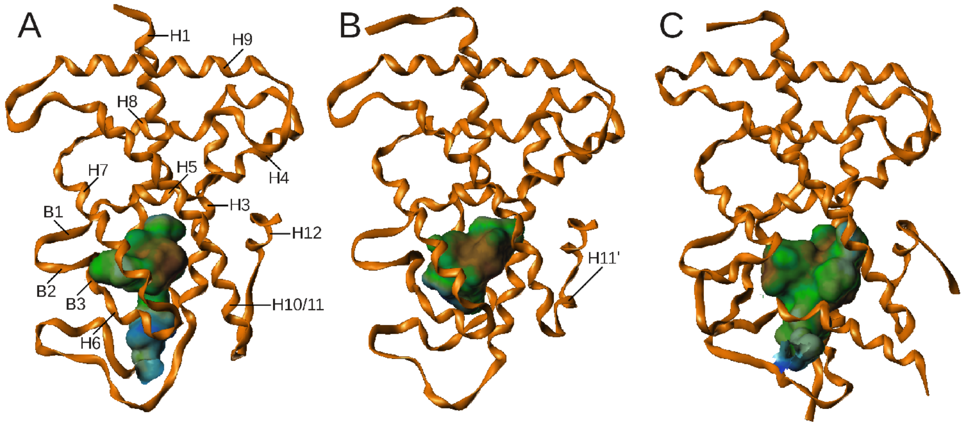

Figure 2. Comparison of ligand-binding domain structures. The course of the protein backbone for hsPXR (A, PDB code 1nrl) and hsCAR (B, PDB code 1xv9) x-ray crystal structures, as well as the xlCARα homology model (C) is displayed using ribbons. The shape of the ligand-binding pocket is indicated using a surface representation. Hydrophobic areas of the LBP are represented in brown, hydrophilic areas are colored in blue.
doi:10.1371/journal.pone.0096263.g002

acid composition is less hydrophobic compared to human CAR and provides more possibilities for sharing hydrogen bonds with ligands. A third of the amino acids constituting the ligand-binding pocket are capable of serving as hydrogen bond donor or acceptor via their side chain and/or backbone atoms (see Table 1).

Basal activity

Based on the homology model structure described above as well as on sequence comparisons with human CAR, we tested the relevance of selected amino acids within the LBD for basal receptor activity by mutating the respective amino acids to their corresponding residues in human CAR (Fig. 3). The selection focused on amino acids lining and surrounding the ligand-binding pocket of which some have been already identified as important for human CAR basal activity. Mutations towards the human ortholog not only provides the opportunity to investigate differences between human and *Xenopus* CAR but it is also beneficial compared to the conventional alanine mutation approach as the small alanine side chain may result in unwanted and non-predictable rearrangements of neighbored amino acids within the LBP [12].

Previously, we have shown a marked increase in basal activity upon mutation of Met272 into valine and of Met316 into phenylalanine, as well as an additive effect of both replacements [3], which was confirmed in the present study. We additionally report here the 8.6-fold increase in basal activity upon mutating Thr305, located on helix 6, into the corresponding human CAR amino acid valine (Val232) (Fig. 3A). The already low basal activity was further decreased 3-fold upon mutation of amino acid Val315 into isoleucine. Not unexpectedly, mutation of Tyr311 into the closely related phenylalanine did not reveal a significant change in basal activity. Also the mutation of His295, predicted to form a hydrogen bond with His233 on helix 3 and located more than 15 Å away from the LBD/H12 interface, did not influence basal activity.

In order to evaluate the molecular basis of the significant increase in basal activity of the three gain-of-function mutants, as well as of the double mutant Met272Val/Met316Phe, we

Table 1. Amino acids constituting the xlCARα ligand-binding pocket.

Location	Amino Acid
H1′	Phe171
H1–H3 loop	Leu231
H3	His233, Phe234, Leu237, Ser238, Met241, Ile242
H5	Met272, Ala275, His276, Phe279
H5–S1 loop	Tyr283
S1–S2 loop	Asn288
S2	Phe290, Cys292
S3	His295, Phe297, Ser298
H6	Ile299, Asp301, Gly302, Ile304, Thr305
H6–H7 loop	Phe307
H7	Tyr311, Leu312, Val315, Met316, Gln319
H10	Tyr399
H12	Met418

Underlined residues provide hydrogen bond acceptor or donors interaction capabilities for ligands via backbone and/or side chain atoms.
doi:10.1371/journal.pone.0096263.t001

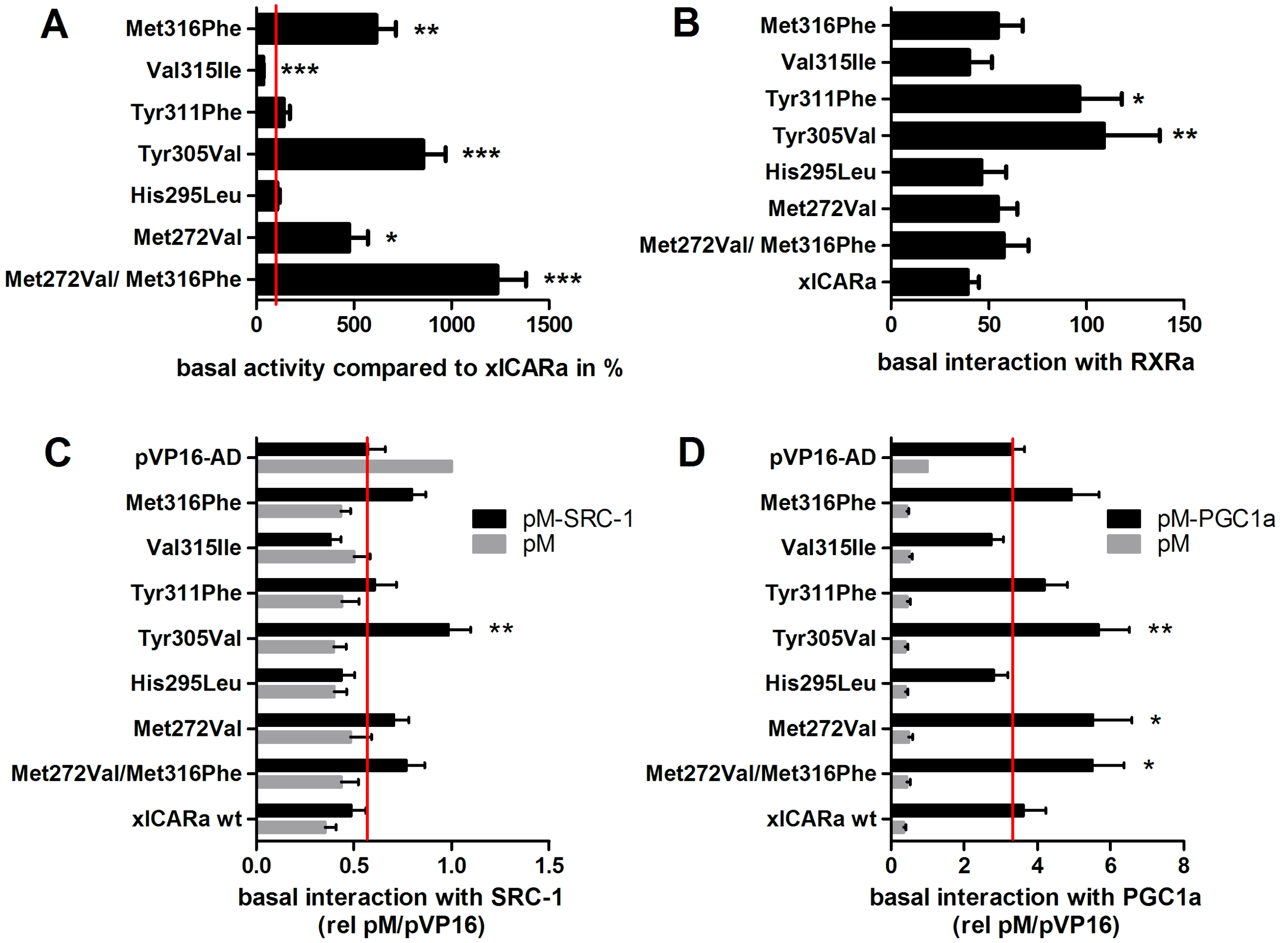

Figure 3. Basal activity of xlCARα mutants and their constitutive interaction with RXRα and coactivators. (A) LS174T cells were co-transfected with the DR4-TK promoter reporter gene plasmid and expression plasmids encoding the indicated xlCARα mutants. Data are presented as % of normalized activity of wild type xlCARα, which was set as 100% and is indicated as a red line. Bars show means ±SEM (n≥8 independent experiments). Statistically significant differences to wild type xlCARα, as determined by one-sample t-tests (corrected for multiple testing by the method of Bonferroni) are indicated by asterisks. (B–D) Mammalian two-hybrid assays were used to analyze the interaction of xlCARα mutants with RXRα (B), SRC-1 (C) and PGC-1α (D). To this end COS7 cells were co-transfected with the GAL4-dependent pGL3G5 reporter, and expression plasmids encoding fusions of VP16-AD and the respective LBD of the indicated xlCARα mutants together with expression plasmids encoding fusions of GAL4-DBD/RXRα-LBD (B), or GAL4-DBD/SRC1-RID (C), or GAL4-DBD/PGC-1α-RID (D). For (B) bars show means ± SEM (n = 6 independent experiments), with activity of cells transfected with empty vector pVP16-AD set as 1. Statistically significant differences to the activity of wild type xlCARα, as identified by one-way ANOVA with Dunnett's multiple comparisons test are indicated by asterisks. For (C) and (D) bars show means ± SEM (n = 9 independent experiments), with activity of cells transfected with the combination of both empty expression plasmids pVP16-AD and pM set as 1. Statistically significant differences to the activity of pVP16-AD and the respective GAL4-DBD/coactivator RID, as identified by two-way ANOVA with Dunnett's multiple comparisons test, are indicated by asterisks. *, $P<0.05$; **, $P<0.01$; ***, $P<0.001$, no asterisks indicate no differences. Red vertical lines in C and D indicate the activity of the empty vector pVP16-AD.
doi:10.1371/journal.pone.0096263.g003

investigated xlCARα heterodimerization with RXRα, as well as recruitment of coactivator proteins SRC-1 and PGC-1α using mammalian two-hybrid assays. Neither the deactivating mutant Val315Ile, nor the non-effective His295Leu revealed any differences in RXRα heterodimerization to wild type receptor (Fig. 3B). Also, neither the two previously identified gain-of-function mutations Met272Val and Met316Phe nor the double mutant, revealed an altered interaction with RXRα. However, a substantial increase in RXRα interaction was observed for the Thr305Val mutant. Interestingly, also the non-effective Tyr311Phe mutation also showed improved heterodimerization (Fig. 3B). Subsequently, interaction studies with coactivators SRC-1 and PGC-1α were performed. In contrast to human CAR1, which showed a strong constitutive interaction with these coactivators [3], wild type xlCARα did not interact constitutively (Fig. 3C&D). A slight but statistically significant constitutive interaction with SRC-1 was only observed for mutant Thr305Val (Fig. 3C). All gain-of-function mutants revealed modest but statistically significant interaction with PGC-1α, with the exception of Met316Phe, where the difference to empty pVP16-AD did not reach statistical significance ($P = 0.12$) (Fig. 3D). With the exception of Met272Val, the enhanced interaction of these mutants with heterodimerization partner RXRα and coactivators SRC-1 and PGC-1α in the mammalian two-hybrid assays was most likely not an artifact of increased protein expression levels of the respective VP16-AD/xlCARα-LBD fusion proteins, as all mutants, with the exception of Met272Val, showed protein expression equal to or even lower (Met316Phe) than wild type (Fig. S3). In contrast, the expression of the fusion protein of Met272Val appeared to be increased, which may explain its enhanced interaction with PGC-1α.

The significantly enhanced interaction of the Thr305Val mutant with both RXRα and PGC-1α prompted us to further investigate this mutation on a molecular level of detail. Thus, we intended to perform comparative molecular dynamics simulations of the wild type and Thr305Val mutant xlCARα protein model. However, due to the largely missing H1-H3 insert, interactions between the truncated H1–H3 region and the Thr305-carrying helix 6 may lead to artificial results. Therefore, we applied a reverse approach in which a human-to-frog mutation (Val232Thr) was introduced into the human CAR x-ray crystal structure (PDB code 1xvp). Analysis of the molecular dynamics trajectories revealed significant differences compared to simulations performed for the wild type receptor published recently [19]. Helices in close proximity of Thr232 were found to be less stable throughout the simulations compared to the wild type receptor. While in one simulation a complete unfolding of helix 6 was observed, a second and third simulation revealed larger rearrangements within the C-terminal region of the LBD (Fig. S4).

Evaluation of the H10/11–H12 region

X-ray crystal structures of human and mouse CAR possess short helices H11′ (also termed helix X) in between helices 10/11 and 12 which has been suggested to represent an important determinant of the high basal activity of mammalian CAR [9]. In contrast, available structures of human PXR show a loop at the corresponding location and our protein model predicts the same for xlCARα. We assessed the importance of length differences of the H10/11–H12 region in human and *Xenopus* CAR by deleting Asp408 and Ile409 from xlCARα. The shortening was accompanied by a further reduction in basal activity (Fig. 4). Conversely, the extension of the H10/11–H12 region of human CAR by inserting aspartate and isoleucine after Gln334 of human CAR reduced the basal activity by more than 50% (Fig. 4). Next, we aimed to clarify the existence of a helical structure in the H10/11–H12 region. Therefore, we mutated Asn413 in the constitutively active xlCARα double-mutant Met316Phe/Met272Val into the helix-breaking residue proline, in order to destroy any potentially existing helical fold in this region. As a proof of concept, we also mutated H11′ amino acid Ala338 of hsCAR into proline. While the constitutive activity of human CAR dropped significantly, that of the xlCARα double mutant did not change (Fig. 4).

Identification of ligand binding modes

In order to investigate the binding modes of structurally diverse xlCARα agonists artemisinin, fenofibrate and 5β-pregnane-3,20-dione (in the following denoted as pregnanedione), three-dimensional structures of the compounds were docked into the ligand-binding pocket of the xlCARα model using the docking program GOLD in combination with the ChemScore scoring function. In order to take ligand efficiency into account, docking scores were corrected as described previously [27]. For pregnanedione only a single binding mode was obtained while for both artemisinin and fenofibrate two slightly differing binding orientations within the LBP were found (data not shown). Due to the barrier between LBP and activation helix, formed by amino acids from helices H3, H5 and H10/11, none of the ligands directly interacted with residues of the activation helix.

Docking poses for artemisinin revealed lowest docking scores of all three ligands (Table 2). The compound is placed in the upper part of the LBP and contacts 10 amino acids (Table 3). Besides extensive van der Waals (vdW) contacts with amino acids of the ligand-binding pocket, the compound shares a hydrogen bond with the Tyr311 side chain (Fig. 5A). An alternative docking pose indicates a hydrogen bond shared with Gln319. Similar to human CAR, both binding modes indicate partial occupation of the S1 sub-pocket, which has been identified as an important area for potent receptor activation [19].

Docking poses for fenofibrate revealed significantly higher docking scores compared to artemisinin (Table 2). The compound interacts with 18 amino acids (Table 3) and occupies a large part of the LBP (Fig. 5B), including the S1 sub-pocket. Most docking poses were found to share a hydrogen bond with His295. Although the compound possesses further hydrogen bond acceptors, no additional H-bond formation with the receptor was observed.

Molecular docking calculations suggest the steroid pregnanedione to bind with highest affinity (Table 2). The ligand contacts 12 amino acids in the LPB via vdW interactions and in addition shares hydrogen bonds with amino acids His233, His295 and Tyr311 (Fig. 5C). The steroid scaffold stacks with the phenyl ring of Phe279 and the compound completely occupies the S1 sub-pocket.

Ligand binding affinity and ligand-dependent receptor activation

In order to validate the predicted binding mode exemplarily for the ligand pregnanedione, we performed CARLA assays in which the induction of the interaction of *in vitro* translated CAR proteins with bacterially expressed SRC-1 RID was used as a measure of ligand binding (Fig. 6A&B). Amino acids shown here to contact pregnanedione (Table 3) and to differ from their counterparts in human CAR were mutated towards the situation in the human receptor. Mutation of each individual amino acid, predicted to contact the ligand, reduced the affinity of *in vitro* ligand binding of the respective mutant receptor, shown by a shift of the dose response curves to the right (Fig. 6B). Especially Phe279Leu, His295Leu, Phe297Tyr and Tyr311Phe mutants demonstrated nearly complete loss of binding. The reduced ligand binding affinity of mutants Leu237Ile (hsCAR: Ile164), Phe279Leu (hsCAR: Leu206), Phe297Tyr (hsCAR: Tyr224) and Val315Ile (hsCAR: Leu242), in which the corresponding hsCAR amino acid residues have been shown to make hydrophobic contacts with pregnanedione [9], suggests that xlCARα binds pregnanedione with higher affinity than hsCAR. CARLA performed with human CAR confirmed this assumption as no significant induction of the interaction with SRC-1 was observed (Fig. 6C). Furthermore, the mutants did not show any significant change in basal interaction with SRC-1 in the absence of ligand, as compared to wild type xlCARα (Fig. 6D), thereby confirming the data obtained with the SRC-1 mammalian two hybrid interaction assay (Fig. 3C). In contrast, human CAR demonstrated a significantly higher level of basal interaction with SRC-1 than xlCARα (Fig. 6D).

Next we analyzed whether the impaired ligand binding, which was observed in the SRC-1 CARLA assay, further translates into lower ligand-dependent induction of the respective receptor transactivation activity in reporter gene assays. Figure 7A shows that the dose response curves of fold induction by pregnanedione of six mutants, all contacting pregnanedione by vdW interactions, were shifted to the right, compared to wild type xlCARα, even if three of them (Phe279Leu, Phe297Tyr, Val315Ile) caught up to wild type at the highest dose of 30 μM. In contrast, the dose response curves of His295Leu and Tyr311Phe mutants, both interacting with pregnanedione via hydrogen bonds, did not show clear dose-dependent differences as compared to wild type (Fig. 7B).

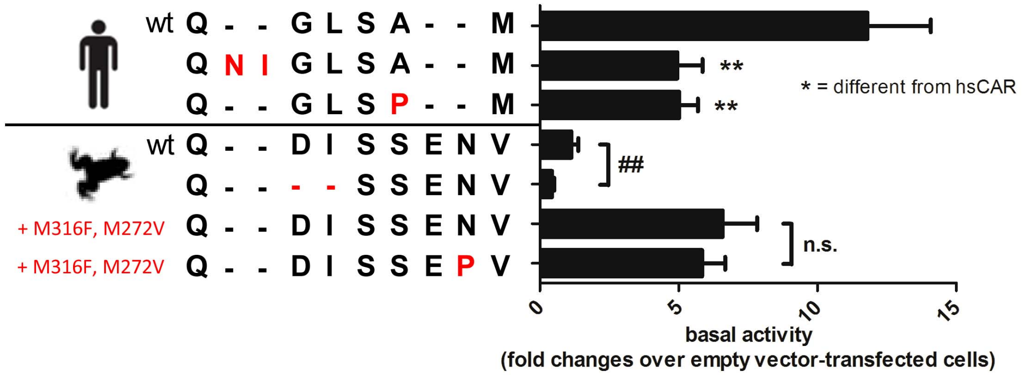

Figure 4. The importance of the H10/11–H12 region for *X.laevis* and human CAR function. Change in receptor basal activity upon deletion or insertion of two amino acids in human and *Xenopus* CAR as well as introduction of the helix-breaking amino acid proline. LS174T cells were co-transfected with the DR4-TK promoter reporter gene plasmid and expression plasmids encoding the indicated CAR proteins. Bars show means ± SEM, with activity of cells transfected with empty expression vector pcDNA3 designated as 1. Statistically significant differences to the activity of the respective wild type CAR were analyzed by one-way ANOVA with Dunnett's multiple comparisons test and indicated by asterisks. *, $P<0.05$; **, $P<0.01$; ***, $P<0.001$.
doi:10.1371/journal.pone.0096263.g004

Discussion

In mammals, nuclear receptors CAR and PXR are key regulators of ligand-dependent expression of detoxifying enzymes and transporter proteins upon exposure of the organism to xenobiotic stress. For several years, the benzoate X receptor has been identified as the only CAR- and PXR-related receptor within *X. laevis*, although with a clearly different physiological function [28]. Recently, we were able to identify two constitutive androstane receptor genes in *X. laevis*, which, in contrast to mammalian CAR, possess a low basal activity and are highly responsive to various compounds [3].

In order to expand our knowledge on these unusual CAR properties in *X. laevis*, we investigated the structural characteristics of the xlCARα ligand-binding domain by constructing a homology model based on sequentially related human CAR and PXR x-ray crystal structures. The selection of our approach is well-founded in the findings of a previous study describing a model of the human CAR LBD utilizing crystallographic data for human VDR and PXR as modeling templates that has been shown to be in very good agreement with published x-ray crystal structures, in particular within the ligand-binding pocket [13].

On the basis of x-ray crystal structures, several structural features have been suggested as important for human CAR basal activity [9]. Besides LBP amino acids directly contacting helix 12, an additional helix (helix 11′) in between helices 10/11 and 12 as well as a salt bridge formed by Lys195 and the C terminus of helix 12 were considered to contribute to the pronounced basal activity. While our model suggests the presence of LBP residues interacting with helix 12, it lacks a helix 11′ like structure, a finding that was confirmed experimentally. Conversely, the importance of helix 11′ for human CAR activity has been verified.

Although a salt bridge between Lys268 and the C terminus was formed (data not shown), similar to the Lys195-C terminus interaction in human CAR, molecular dynamics simulations of human CAR suggest the salt bridge to be instable [20].

Motivated by our recent finding whereupon the low basal activity of xlCARα was substantially increased by mutating just a single amino acid of the LBD towards human CAR, we expanded our mutation analysis guided by the protein model of the *Xenopus* CAR LBD. Moreover, by including two-hybrid assays, the effect on protein-protein interactions with heterodimerization partner RXRα and coactivators SRC-1 and PGC-1α upon introduction of humanizing mutations was investigated. Including the previously reported results, we now have identified altogether three amino acids located on helices H5 (Met272Val), H6 (Thr305Val) and H7 (Met316Phe), whose mutation towards hsCAR revealed a substantial gain-of-function that reached or even exceeded basal activity levels measured for the human receptor.

Initially [3], based on luciferase activity data and the protein model, the structural basis for the increased constitutive activity had been suggested to rely on improved interactions with the activation helix and therefore better coactivator binding (Met272-Val) or enhanced heterodimerization with RXRα (Met316Phe). While the two-hybrid data did not confirm increased interaction with RXRα of these mutants, they suggest significant basal interaction with the coactivator PGC-1α, which was not observed for the wild type. However, the effects appeared to be small and in the case of Met272Val may result from an increased expression of the respective VP16-AD fusion protein. Therefore, the molecular basis for the significantly increased basal activity of Met272Val and Met316Phe remains unknown.

In contrast to these mutations, humanizing Thr305 to the more hydrophobic amino acid valine revealed both reinforced RXRα heterodimerization and enhanced PGC-1α recruitment. Molecular dynamics simulations of a reversed mutation inserted into the hsCAR LBD suggested a partially instable LBD fold upon introduction of a polar amino acid. According to the protein model, Thr305 is located at the very C-terminal end of helix 6 with its polar side chain buried within the protein interior. Mutation into the corresponding human amino acid (Val232) may disrupt hydrogen bond formation with Asp301 on helix 6 which is observed in the protein model. Amino acid side chains in close proximity to Thr305 are either aromatic or aliphatic (Leu231, Phe234, Phe297, Phe307, Ile406), thus the non-polar side chain of the mutant may provide better vdW interaction possibilities with closely neighbored amino acids on helices 3, 6 and 10/11 and thereby stabilize the overall receptor fold.

Tyr311 is predicted to be located at the N terminus of helix 7 with its side chain pointing into the ligand-binding pocket, thus providing an anchor point for bound ligands via its hydroxyl group. As the surrounding amino acid side chains are either of aromatic or aliphatic nature, mutation into phenylalanine may ameliorate vdW contacts with closely neighbored residues and

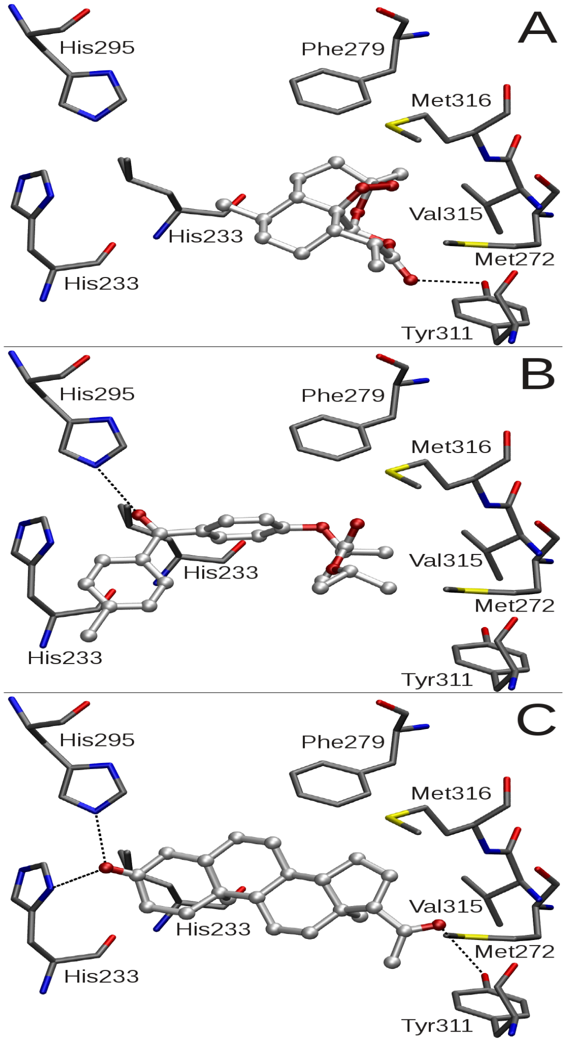

Figure 5. Binding modes of xlCARα agonists within the receptor ligand-binding pocket. Favorable docking poses for artemisinin (A), fenofibrate (B) and pregnanedione (C) within the ligand-binding pocket (LBP) of xlCARα were determined using docking program GOLD. Selected amino acids of the LBP are shown as capped sticks while ligands are displayed in ball and stick representation. Hydrogen bonds between ligands and protein are shown as dotted lines.
doi:10.1371/journal.pone.0096263.g005

thereby stabilize the local fold of the receptor which includes the heterodimerization motif (helix 10/11), thus providing an explanation for the experimentally observed improvement in RXRα binding. The unaltered basal activity is probably due to the unchanged PGC-1α coactivator binding.

Mutation of Val315 towards isoleucine may sterically slightly reorient the neighbored amino acid Tyr311. Using MD simulations, a mutation of the corresponding human CAR amino acid Phe238 to alanine has shown to result in reorientation of the closely neighbored Tyr326 (xlCARα: Tyr399) away from helix 12 into the LBP that may impair stabilization of the activation helix [12]. Therefore, loss of interactions between Tyr311 and Tyr399 may result in decreased basal activity. Of all mutants tested, Val315Ile revealed least interactions with RXRα and coactivator proteins. Although not statistically significant, the observed trend may explain the loss in basal activity.

Pregnanedione, one of the most potent known xlCARα agonists [3], is predicted to be tightly anchored within the LBP via several hydrogen bonds, thus providing a convincing explanation for its extraordinary induction potential. The binding mode suggests the compound to act as a cross-linking agent of several secondary structural elements within the LBD, namely helices 3 and 11 as well as the β_3-strand, thereby stabilizing the overall LBD fold which in turn facilitates heterodimerization with RXRα and coactivator binding. While pregnanedione is a highly potent xlCARα agonist, the compound revealed a modest 2-fold activation in human receptor [29]. Based on an x-ray crystal structure of human CAR a hydrogen bond between pregnanedione and His203 (His276 in xlCARα) has been reported [9]. However, molecular dynamics simulations did not reveal the presence of such an interaction [13]. Instead, the ligand was found to contact the receptor exclusively by vdW interactions and thus is expected to bind with significantly lower affinity to hsCAR than to xlCARα.

Both fenofibrate and artemisinin are less potent agonists, a result that was confirmed by molecular docking calculations. The docking poses suggest that both ligands share only a single hydrogen bond with the receptor and thus bind with lower affinity compared to pregnanedione. While the docked fenofibrate almost completely occupies the LBP and thereby forms many more vdW interactions with amino acids of the ligand-binding pocket than artemisinin, the better activation potential of fenofibrate can be explained by a higher receptor affinity.

Docking predictions were exemplarily confirmed for the pregnanedione by determining ligand binding affinities of receptor mutants *in vitro* using the SRC-1 CARLA assay. Mutation of any single amino acid predicted to contact pregnanedione resulted in markedly impaired ligand binding. Mutations of the two H-bonding amino acids His295 and Tyr311 were among the four most affected in the SRC-1 CARLA assay. In contrast, mutation of these two amino acid residues did not clearly affect ligand-dependent transactivation potential of the respective receptor mutants, which may indicate that they may still be able to recruit coactivators other than SRC-1. Furthermore, this demonstrates the limitations of indirect methods, such as CARLA, for the analysis of ligand binding affinity, which seems to be influenced by the coactivator used. The mutation of any other amino acid,

Table 2. Docking scores for ligand binding poses determined with program GOLD.

Ligand	ChemScore	# of Heavy Atoms	Corr. ChemScore
Artemisinin	27.76	20	6.21
Fenofibrate	36.69	25	7.34
Pregnanedione	37.76	23	7.87

Corrected scores, taking into account the different size of the structures (number of heavy atoms) were determined as described before (26).
doi:10.1371/journal.pone.0096263.t002

Table 3. Protein-ligand interactions between xlCARα and docked agonists.

Location	Artemisinin	Fenofibrate	Pregnanedione
H1′		Phe171	Phe171
H1–H3 loop		Gln177	
H1–H3 loop		Leu182	Leu182
H1–H3 loop		Leu231	
H3		His233	His233
H3	Phe234	Phe234	Phe234
H3		Leu237	Leu237
H3			Ser238
H3	Met241	Met241	
H5	Met272	Met272	Met272
H5	His276		His276
H5	Phe279	Phe279	Phe279
S2		Phe290	
S3		His295	His295
S3	Phe297	Phe297	Phe297
H6		Thr305	
H6–H7 loop	Phe307	Phe307	Phe307
H7	Tyr311	Tyr311	Tyr311
H7	Leu312	Leu312	Leu312
H7		Val315	Val315
H10	Tyr399		

For each agonist docked into the ligand-binding pocket of xlCARα the contacting amino acids (distance criterion of 4.0 Å) and their location on secondary structural elements of the ligand-binding domain is given. Amino acids sharing hydrogen bonds with docked ligands are underlined.
doi:10.1371/journal.pone.0096263.t003

except His295 and Tyr311, contacting pregnanedione did also impair the ligand-dependent transactivation potential of the respective receptors, thereby further confirming molecular docking predictions as well as CARLA results.

His295 and Tyr311 each interact with a single ligand oxygen atom located at both ends of the steroid scaffold, thereby the mutation into the corresponding amino acid in human CAR may less impact ligand binding compared to mutations such as Phe297 with the phenyl side chains extensively contacting A, B and C ring of the compound. Moreover, a new hydrogen bond with His276, located in close proximity of Tyr311 might be formed. While in wild type receptor His276 interacts with pregnanedione only via vdW contacts, a mutated receptor may result in small rearrangements within the LBP, thereby allowing hydrogen bonding and neutralizing the loss of binding affinity resulting from removal of a hydrogen bonding partner. Docking pregnanedione into receptors carrying either the His295Leu or Tyr311Phe mutation revealed the ligand to be placed closer to His276 so that only a small imidazole reorientation is required to form a strong hydrogen bond with the ligand (data not shown).

Compared with all known human CAR splice variants, xlCARα is most similar to splice variant 2 (SV2, UniProt identifier Q14994-8), which contains a five amino acid insert (sequence: APYLT) after Pro270, located on the H8–H9 loop [23]. In contrast to the SV1 isoform (UniProt identifier Q14994-2), often considered as the wild type splice variant, this isoform possesses a low basal activity and substantial responsiveness to CAR ligands [30]. Although hepatic mRNA levels of both isoforms have been shown to reach similar levels [31], [32], research has concentrated mainly on the SV1 isoform. The significant reduction of the xlCARα ligand-dependent activation upon introduction of gain-of-function mutation suggests that the high activity of human CAR-SV1 masks the inducing effects of binding agonists which can be exposed by introducing just a single amino acid of the APYLT motif [33].

Considering also the structural information derived from our homology model as well as the accompanying assays, *X. laevis* CARα clearly more resembles the mammalian pregnane X receptor rather than mammalian CAR. Compared to human CAR (SV1 and SV2 isoforms), the ligand-binding pocket appears to be substantially larger and provides significantly more possibilities for establishing hydrogen bonds with bound ligands. Shaped similar to the hsPXR LBP, the xlCARα LBP may also harbor larger and chemically more diverse ligands as human CAR. Previously, we have shown that SR12813 (MW 505 Da), initially identified as human PXR inducer [34], is a potent xlCARα agonist [3]. By contrast, the compound does not induce human CAR activity [3], [35]. As predicted by the protein model and confirmed by mutational studies, the *X. laevis* constitutive androstane receptor does not possess a helical secondary structural element in between helices 10/11 and 12 as found in human CAR x-ray crystal structures of splice variant 1 (H11′) but rather is expected to possess a PXR-like loop conformation. Moreover, our results experimentally confirm the important role of H11′ in hsCAR basal activity.

Similar to PXR and also the related vitamin D receptor (VDR, NR1I1), xlCARα possesses a long H1–H3 insert. However, this part of the LBD is not involved in the LBP formation as shown for PXR and therefore is not expected to affect the fold of helices 6 and 7 as observed in all PXR x-ray crystal structures [36]. The

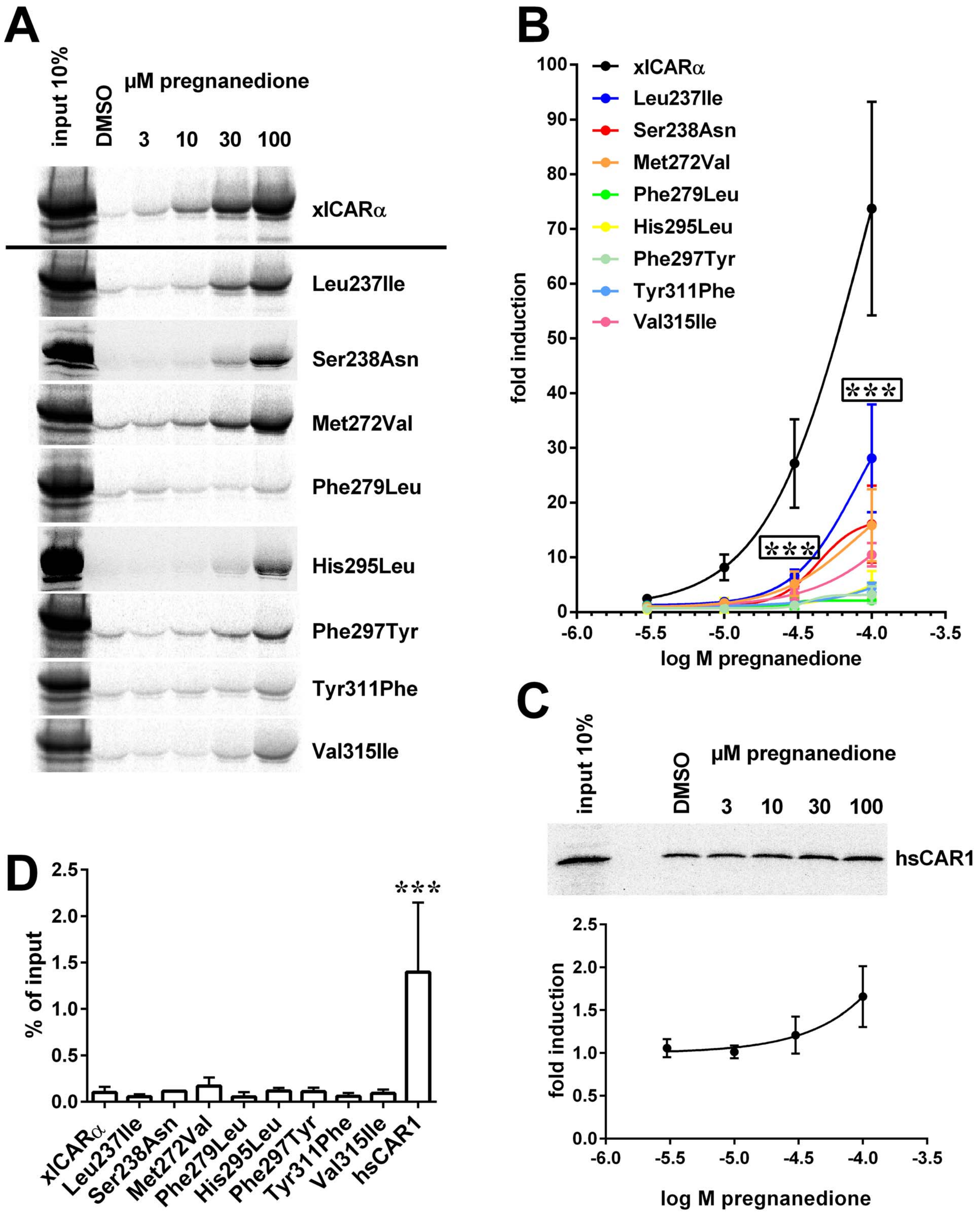

Figure 6. Constitutive interaction and ligand-dependent induction of the interaction of xlCARα mutants with coactivator SRC-1. CARLA assays were performed using bacterially expressed GST-SRC-1 RID protein and *in vitro* translated ^{35}S-labeled xlCARα wild type or mutant proteins or human CAR1 protein (hsCAR1), respectively, in the presence of the indicated concentrations of pregnanedione or solvent only (DMSO). (A) The results of representative experiments with xlCARα wild type and mutant proteins are shown. (B,C) Dose response analysis of ligand-dependent induction of SRC-1 interaction of xlCARα mutants (B) and human CAR1 (C), with graphs showing means ± SD (n = 3 independent experiments) of fold induction by respective concentrations of pregnanedione. Activity in the presence of DMSO only was designated as 1. In the upper part of (C), a representative experiment with human CAR1 is shown. Data were analyzed by two-way ANOVA with Dunnett's multiple comparisons test (B) or one sample t-test (C). Significant differences to wild type xlCARα are shown by asterisks. The boxed asterisks indicate significant difference for all mutants at the respective dose. (D) Columns show means ± SD (n = 3 independent experiments) of the constitutive interaction with SRC-1 in the absence of ligand. Data were analyzed by one-way ANOVA with Dunnett's multiple comparisons test. Significant difference to wild type xlCARα is shown by asterisks. ***, $P < 0.001$.
doi:10.1371/journal.pone.0096263.g006

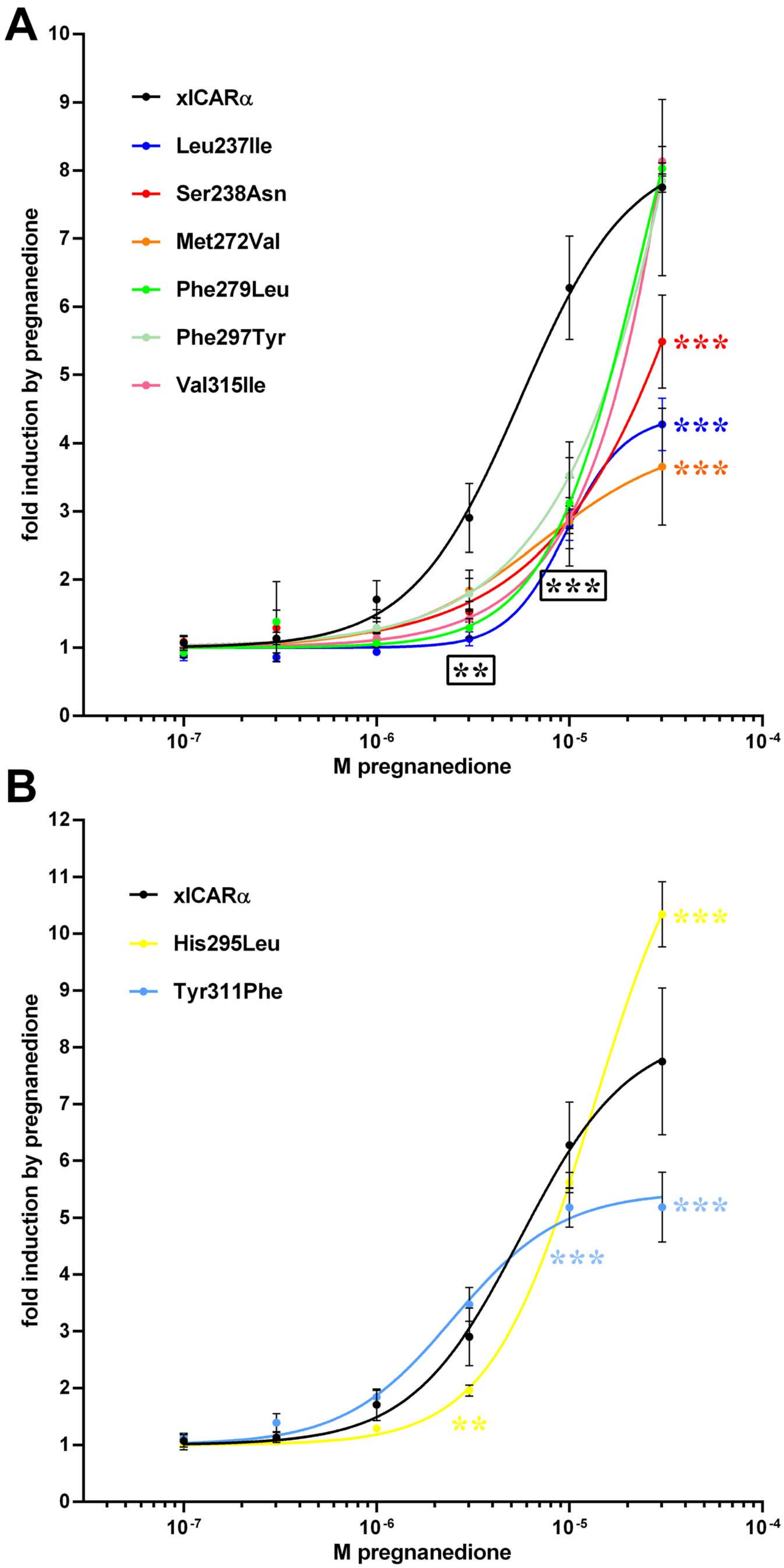
A
xlCARα
Leu237Ile
Ser238Asn
Met272Val
Phe279Leu
Phe297Tyr
Val315Ile
fold induction by pregnanedione
M pregnanedione
10^-7
10^-6
10^-5
10^-4
B
xlCARα
His295Leu
Tyr311Phe
fold induction by pregnanedione
M pregnanedione

Figure 7. Dose-reponse analysis of ligand-dependent activities of xlCARα mutants. HepG2 cells were co-transfected with expression plasmids encoding wild type xlCARα or the indicated mutants and DR4-TK promoter reporter gene and treated with pregnanedione as indicated. Graphs show means ± SD (n≥3 independent experiments) of fold induction by respective concentrations of pregnanedione, with activity in the presence of solvent DMSO only set as 1. (A) presents the dose response curves of xlCARα proteins with mutations of amino acid residues contacting pregnanedione via vdW interactions, whereas (B) presents the respective dose response curves of xlCARα proteins with mutations of amino acid residues contacting pregnanedione by H-bonds, each in comparison to the same dose response curve of wild type xl CARα. Data were analyzed by two-way ANOVA with Dunnett's multiple comparisons test. Significant differences to wild type xlCARα are shown by asterisks, whereby colors refer to the respective mutants. Black asterisks in a box indicate significant difference for all mutants at the respective dose. **, $P<0.01$; ***, $P<0.001$.
doi:10.1371/journal.pone.0096263.g007

deletion of a homologous region from hsVDR and xlVDR had also no effect on the selectivity for bile acids of these receptors [37]. Therefore, the functional role of the H1–H3 insert in xlCARα still remains to be elucidated.

Taken together, our study provides the first in-depth view into the structural and functional characteristics of a non-mammalian xenosensor that suggests its structural and functional similarity to the mammalian pregnane X receptor using a combination of computational and biochemical methods. In addition, our approach proved to be a procedure for investigating the contribution of single amino acids to basal activity of human CAR. Due to the ligand promiscuity of the receptor and to its wide organ distribution, exogenous xlCARα agonists might be problematic as environmental pollution increases. Therefore, our study may represent a preliminary step for the development of an *in silico* assessment of chemicals for unwanted effects in amphibians.

Supporting Information

Figure S1 Sequence alignment of xlCARα template structure generated using CLUSTALW. Secondary structure prediction for xlCARα (H, helix; E, strand; C, coil) was performed using PSIPRED (http://bioinf.cs.ucl.ac.uk/psipred/). Predictions for single amino acids with a confidence level higher than 5 are highlighted in bold.
(TIF)

Figure S2 Equal expression of *in vitro* synthesized CAR proteins. Wild type and mutant xlCARα, and hsCAR1 proteins were labeled with ^{35}S-methionine by in vitro transcription/translation using the respective expression plasmids. Aliquots of the reactions were analyzed by protein gel electrophoresis.
(TIF)

Figure S3 Expression levels of CAR-VP16 fusion proteins in COS 7 cells. Expression plasmids encoding fusion proteins of VP16-AD with wild type or mutant xlCARα or hsCAR LBD used in the mammalian two-hybrid assays were cotransfected with a *Renilla* luciferase reporter gene plasmid. Protein expression was analyzed in Western blots with anti-VP16 antibody and normalized for transfection efficiency via *Renilla* luciferase activity. The upper panel shows the respective quantification of VP16-AD/CAR-LBD fusion protein expression as mean ± SEM of 2 independent experiments. The two respective Western Blots are shown in a) and b).
(TIF)

Figure S4 Conformational rearrangements within the ligand-binding domain of hsCAR upon the introduction of a Val232Thr mutation. Structures of wildtype (mauve) and mutant (yellow) receptor emerging after 50 ns of 3 different unconstrained molecular dynamics simulations are superimposed on the hsCAR x-ray crystal structure (PDB code 1xvp, colored in green). For the sake of clarity the structures are shown in ribbon representation and large parts of the LBD are excluded. The side chain of Val232 of the x-ray crystal structure is shown explicitly (green).
(TIF)

Table S1 Primers used for generation of point mutations and deletions within the hsCAR, xlCARα and hsPXR ligand-binding domains.
(DOCX)

Author Contributions

Conceived and designed the experiments: MM CN OB LW BW. Performed the experiments: MM CN OB UG BW. Analyzed the data: MM CN OB UG HH BW. Contributed reagents/materials/analysis tools: OB LW BW. Wrote the paper: OB HH LW BW.

References

1. Hwang-Verslues WW, Sladek FM (2010) HNF4α – role in drug metabolism and potential drug target? Curr Opin Pharmacol 10: 698–705.
2. Tolson AH, Wang H (2010) Regulation of drug-metabolizing enzymes by xenobiotic receptors: PXR and CAR. Adv Drug Deliv Rev 38: 2091–2095.
3. Mathäs M, Burk O, Qiu H, Nußhag C, Gödtel-Armbrust U, et al. (2012) Evolutionary history and functional characterization of the amphibian xenosensor CAR. Mol Endocrinol 26: 14–26.
4. di Masi A, De Marinis E, Ascenzi P, Marino M (2009) Nuclear receptors CAR and PXR: Molecular, functional, and biomedical aspects. Mol Aspects Med 30: 297–343.
5. Wang YM, Ong SS, Chai SC, Chen T (2012) Role of CAR and PXR in xenobiotic sensing and metabolism. Expert Opin Drug Metab Toxicol 8: 803–817.
6. Baes M, Gulick T, Choi HS, Martinoli MG, Simha D, et al. (1994) A new orphan member of the nuclear hormone receptor superfamily that interacts with a subset of retinoic acid response elements. Mol Cell Biol 14: 1544–1552.
7. Choi HS, Chung M, Tzameli I, Simha D, Lee YK, et al. (1997) Differential transactivation by two isoforms of the orphan nuclear hormone receptor CAR. J Biol Chem 272: 23565–23571.
8. Dussault I, Lin M, Hollister K, Fan M, Termini J, et al. (2002) A structural model of the constitutive androstane receptor defines novel interactions that mediate ligand-independent activity. Mol Cell Biol 22: 5270–5280.
9. Xu RX, Lambert MH, Wisely BB, Warren EN, Weinert EE, et al. (2004) A structural basis for constitutive activity in the human CAR/RXRα heterodimer. Mol Cell 16: 919–926.
10. Frank C, Molnár F, Matilainen M, Lempiäinen H, Carlberg C (2004) Agonist-dependent and agonist-independent transactivations of the human constitutive androstane receptor are modulated by specific amino acid pairs. J Biol Chem 279: 33558–33566.
11. Jyrkkärinne J, Windshügel B, Mäkinen J, Ylisirniö M, Peräkylä M, et al. (2005) Amino acids important for ligand specificity of the human constitutive androstane receptor. J Biol Chem 280: 5960–5971.
12. Windshügel B, Jyrkkärinne J, Poso A, Honkakoski P, Sippl W (2005) Molecular dynamics simulations of the human CAR ligand-binding domain: deciphering the molecular basis for constitutive activity. J Mol Model 11: 69–79.
13. Windshügel B, Jyrkkärinne J, Vanamo J, Poso A, Honkakoski P, et al. (2007) Comparison of homology models and X-ray structures of the nuclear receptor CAR: assessing the structural basis of constitutive activity. J Mol Graph Model 25: 644–657.
14. Berman HM, Battistuz T, Bhat TN, Bluhm WF, Bourne PE, et al. (2002) The Protein Data Bank. Acta Crystallogr D Biol Crystallogr 58: 899–907.
15. Watkins RE, Wisely GB, Moore LB, Collins JL, Lambert MH, et al. (2001) The human nuclear xenobiotic receptor PXR: structural determinants of directed promiscuity. Science 292: 2329–2333.

16. Laskowski RA, MacArthur MW, Moss DS, Thornton JM (1993) PROCHECK - a program to check the stereochemical quality of protein structures. J Appl Cryst 26: 283–291.
17. Sippl MJ (1993) Recognition of Errors in Three-Dimensional Structures of Proteins. Proteins 17: 355-362.
18. Canutescu AA, Shelenkov AA, Dunbrack RL Jr (2003) A graph-theory algorithm for rapid protein side-chain prediction. Protein Sci 12: 2001–2014.
19. van der Spoel D, Lindahl E, Hess B, Groenhof G, Mark AE, et al. (2005) GROMACS: Fast, flexible and free. J. Comput. Chem. 26: 1701–1718.
20. Windshügel B, Poso A (2011) Constitutive activity and ligand-dependent activation of the nuclear receptor CAR—insights from molecular dynamics simulations. J Mol Recognit 24: 875–882.
21. Hustert E, Zibat A, Presecan-Siedel E, Eiselt R, Mueller R, et al. (2001) Natural protein variants of pregnane X receptor with altered transactivation activity toward CYP3A4. Drug Metab Dispos 29: 1454–1459.
22. Geick A, Eichelbaum M, Burk O (2001) Nuclear receptor response elements mediate induction of intestinal MDR1 by rifampin. J Biol Chem 276: 14581–14587.
23. Arnold KA, Eichelbaum M, Burk O (2004) Alternative splicing affects the function and tissue-specific expression of the human constitutive androstane receptor. Nucl Recept 2: 1.
24. Bros M, Ross XL, Pautz A, Reske-Kunz AB, Ross R (2003) The human fascin gene promoter is highly active in mature dendritic cells due to a stage-specific enhancer. J. Immunol. 171: 1825–1834.
25. Burk O, Tegude H, Koch I, Hustert E, Wolbold R, et al. (2002) Molecular mechanisms of polymorphic CYP3A7 expression in adult human liver and intestine. J Biol Chem 277: 24280–24288.
26. Gödtel-Armbrust U, Metzger A, Kroll U, Kelber O, Wojnowski L (2007) Variability in PXR-mediated induction of CYP3A4 by commercial preparations and dry extracts of St. John's wort. Naunyn Schmiedebergs Arch Pharmacol 375: 377–382.
27. Burk O, Piedade R, Ghebreghiorghis L, Fait JT, Nussler AK, et al. (2012) Differential effects of clinically used derivatives and metabolites of artemisinin in the activation of constitutive androstane receptor isoforms. Br J Pharmacol 167: 666–681.
28. Grün F, Venkatesan RN, Tabb MM, Zhou C, Cao J, et al. (2002) Benzoate X receptors alpha and beta are pharmacologically distinct and do not function as xenobiotic receptors. J Biol Chem 277: 43691–43697.
29. Moore LB, Parks DJ, Jones SA, Bledsoe RK, Consler TG, et al. (2000) Orphan nuclear receptors constitutive androstane receptor and pregnane X receptor share xenobiotic and steroid ligands. J. Biol. Chem. 275: 15122–15127.
30. Auerbach SS, Stoner MA, Su S, Omiecinski CJ (2005) Retinoid X receptor-alpha-dependent transactivation by a naturally occurring structural variant of human constitutive androstane receptor (NR1I3). Mol Pharmacol 68: 1239–1253.
31. Jinno H, Tanaka-Kagawa T, Hanioka N, Ishida S, Saeki M, et al. (2004) Identification of novel alternative splice variants of human constitutive androstane receptor and characterization of their expression in the liver. Mol Pharmacol 65: 496–502.
32. Ross J, Plummer SM, Rode A, Scheer N, Bower CC, et al. (2010) Human constitutive androstane receptor (CAR) and pregnane X receptor (PXR) support the hypertrophic but not the hyperplastic response to the murine nongenotoxic hepatocarcinogens phenobarbital and chlordane in vivo. Toxicol Sci 116: 452–466.
33. Chen T, Tompkins LM, Li L, Li H, Kim G, et al. (2010) A single amino acid controls the functional switch of human constitutive androstane receptor (CAR) 1 to the xenobiotic-sensitive splicing variant CAR3. J Pharmacol Exp Ther 332: 106–115.
34. Jones SA, Moore LB, Shenk JL, Wisely GB, Hamilton GA, et al. (2000) The pregnane X receptor: a promiscuous xenobiotic receptor that has diverged during evolution. Mol Endocrinol 14: 27–39.
35. Moore LB, Maglich JM, McKee DD, Wisely B, Willson TM, et al. (2002) Pregnane X receptor (PXR), constitutive androstane receptor (CAR), and benzoate X receptor (BXR) define three pharmacologically distinct classes of nuclear receptors. Mol Endocrinol 16: 977–986.
36. Carnahan VE, Redinbo MR (2005) Structure and function of the human nuclear receptor PXR. Curr Drug Metab 6: 357–367.
37. Krasowski MD, Ai N, Hagey LR, Kollitz EM, Kullman SW, et al. (2011) The evolution of farnesoid X, vitamin D, and pregnane X receptors: insights from the green-spotted pufferfish (Tetraodon nigriviridis) and other non-mammalian species. BMC Biochem 12: 5.
